# Supplementary material for: Rp3: Ribosome profiling-assisted proteogenomics improves coverage and confidence during microprotein discovery
Source: Nat Commun. 2024 Aug 9;15:6839. doi: 10.1038/s41467-024-50301-4 (PMC11316118; doi:10.1038/s41467-024-50301-4)
Supplement: Supplementary file 1 — Supplementary Information [file 41467_2024_50301_MOESM1_ESM.docx]

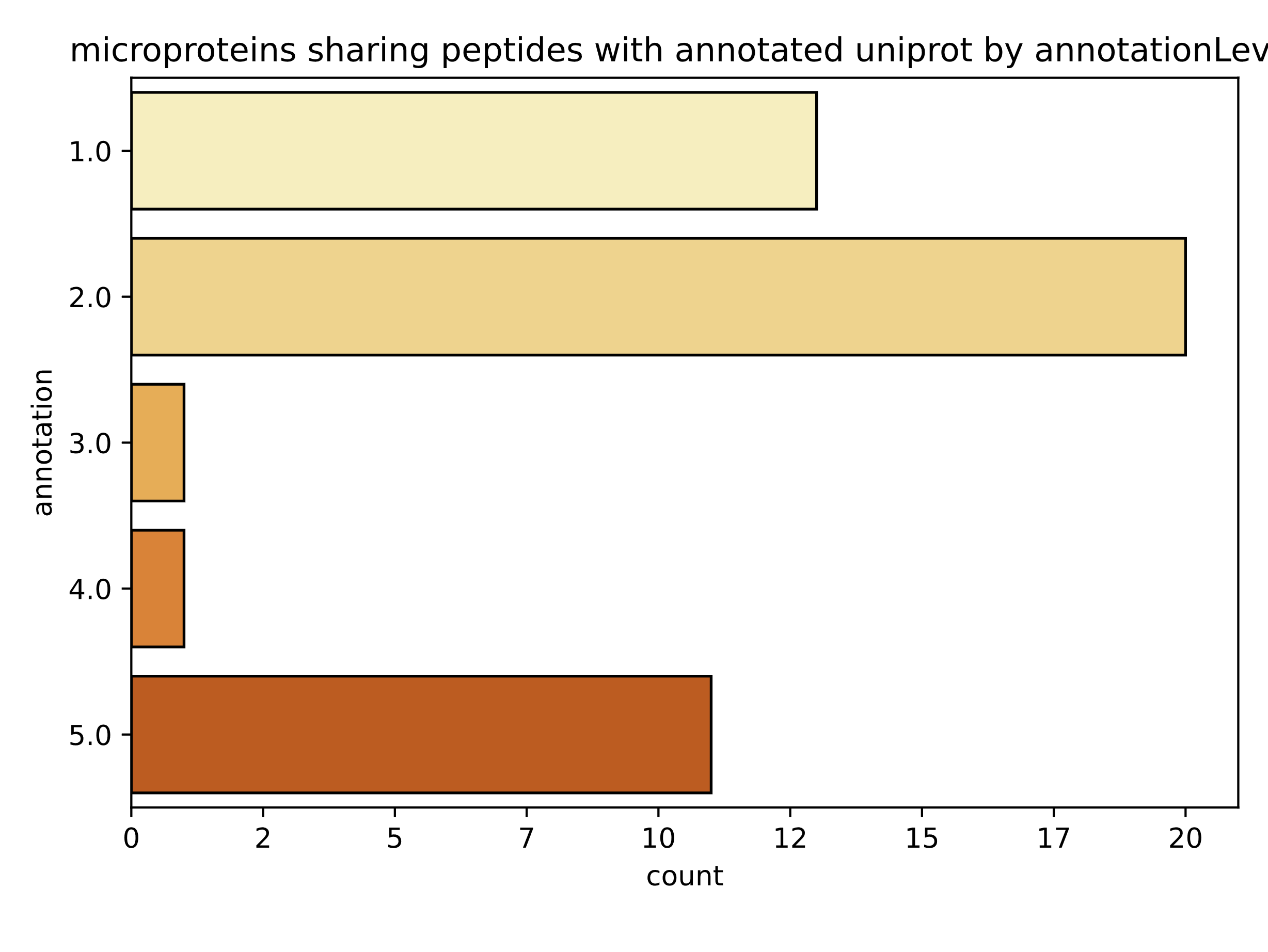


**Supplementary Figure 1.** Number of mass spectrometry microproteins identified with Rp3 for the mouse dataset with at least one peptide that also matched a protein in the extended, unreviewed Uniprot database, grouped by the annotation level of the matched protein.


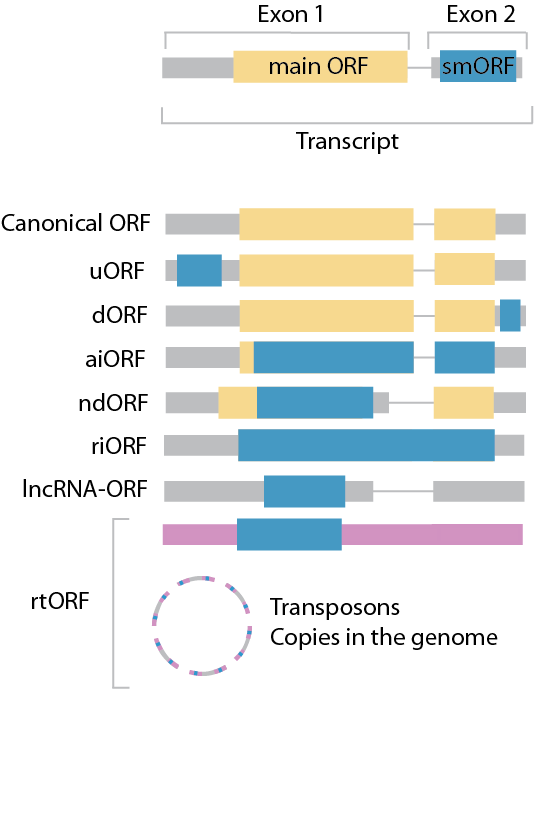


**Supplementary Figure 2.** smORF type classification based on genomic features overlapped in the Ensembl annotation. **Canonical ORF**: previously annotated ORF, in these cases sharing a transcript with an unannotated smORF. **uORF**: smORF present upstream of an annotated ORF. **dORF**: smORF present downstream from an annotated ORF. **aiORF:** smORF with an alternative initiation site with respect to the annotated ORF. **ndORF:** non-defined ORF within an alternatively spliced transcript, as defined by the Ensembl annotation (https://useast.ensembl.org/info/genome/genebuild/biotypes.html). **riORF**: smORF spanning across a retained intron. **lncRNA-ORF**: smORF present in a transcript previously annotated as long non-coding RNA (lncRNA). **rtORF**: smORF present in a pseudogene or retrotransposon.


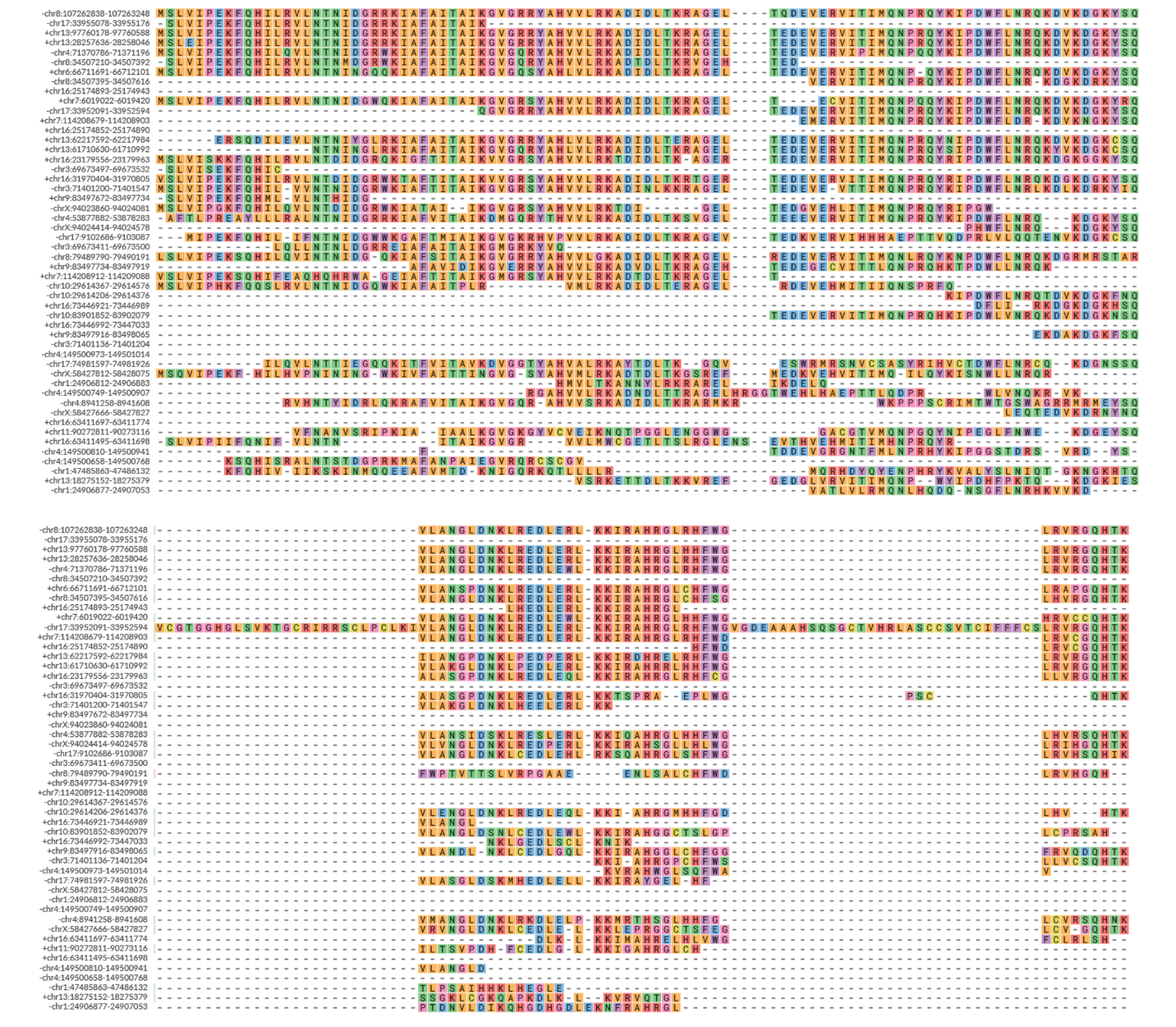


**Supplementary Figure 3.** Complete multiple sequence alignment of the representative smORF-chr8_107262838-107263248 (Fig. 4) and all its homologous sequences in the genome identified by tBlastn.


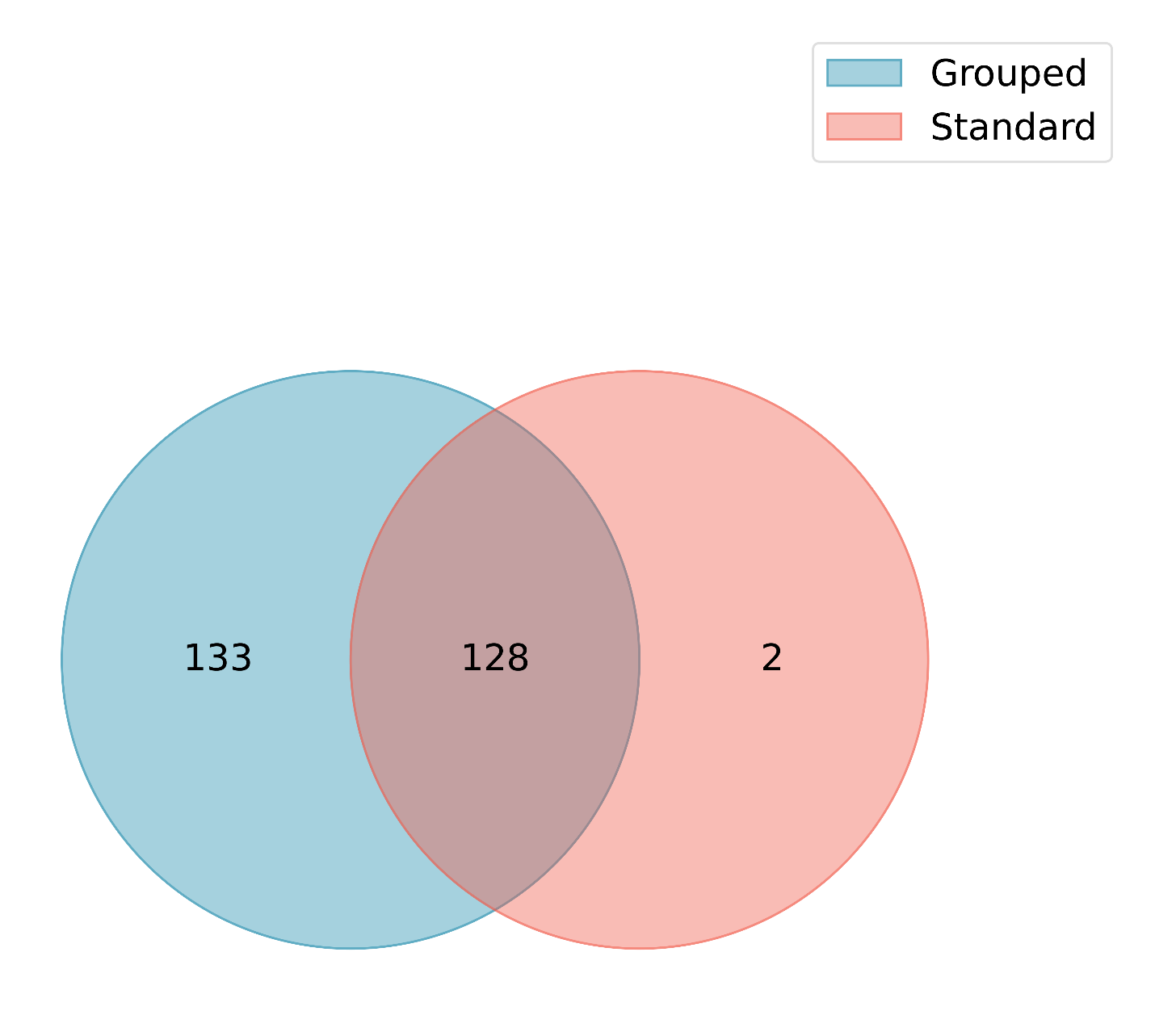


**Supplementary Figure 4.** Venn diagram showing the overlap between the Rp3 results for mouse datasets where FDR was assessed with canonical proteins and unannotated microproteins together (Standard), and results where canonical proteins and unannotated microproteins were grouped separately and FDR was assessed once for each group (Grouped).


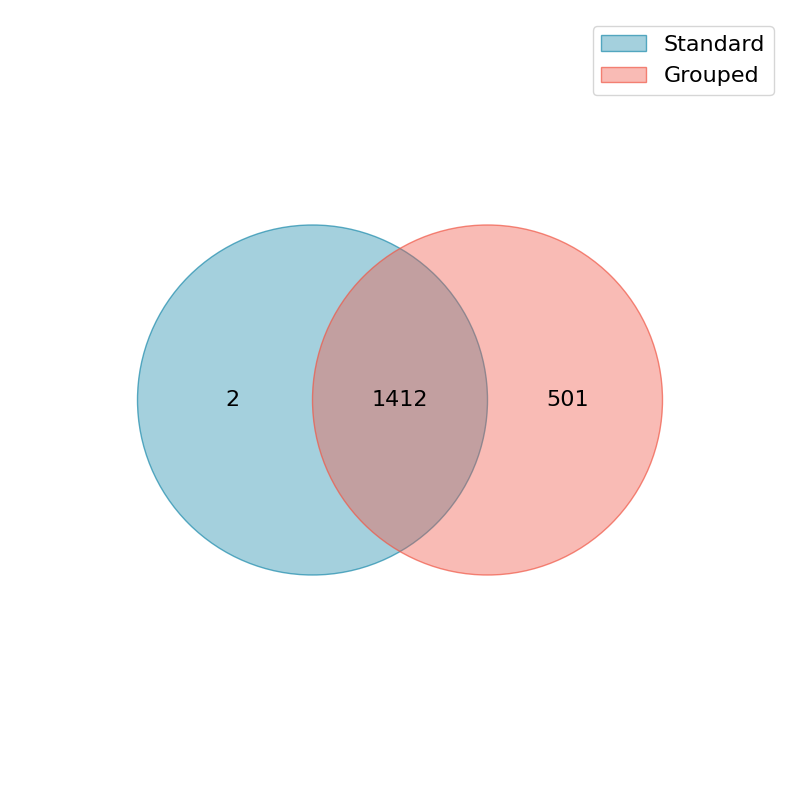


**Supplementary Figure 5.** Venn diagram showing the overlap between the Rp3 results for human datasets where FDR was assessed with canonical proteins and unannotated microproteins together (Standard), and results where canonical proteins and unannotated microproteins were grouped separately and FDR was assessed once for each group (Grouped). These results were not filtered to include only predictions from mhc-flurry; thus they contain direct results from the Rp3 pipeline.
