# Supplementary material for: Rp3: Ribosome profiling-assisted proteogenomics improves coverage and confidence during microprotein discovery
Source: Nat Commun. 2024 Aug 9;15:6839. doi: 10.1038/s41467-024-50301-4 (PMC11316118; doi:10.1038/s41467-024-50301-4)
Supplement: Supplementary file 2 — Reporting Summary [file 41467_2024_50301_MOESM2_ESM.pdf]

nature portfolio

Corresponding author(s): Dr. Alan Saghatelian

Last updated by author(s): Jun 14, 2024

Reporting Summary

Nature Portfolio wishes to improve the reproducibility of the work that we publish. This form provides structure for consistency and transparency in reporting. For further information on Nature Portfolio policies, see our Editorial Policies and the Editorial Policy Checklist.

Statistics

For all statistical analyses, confirm that the following items are present in the figure legend, table legend, main text, or Methods section.

n/a	Confirmed	
☐	☒	The exact sample size (*n*) for each experimental group/condition, given as a discrete number and unit of measurement
☐	☒	A statement on whether measurements were taken from distinct samples or whether the same sample was measured repeatedly
☐	☒	The statistical test(s) used AND whether they are one- or two-sided<br>*Only common tests should be described solely by name; describe more complex techniques in the Methods section.*
☐	☒	A description of all covariates tested
☐	☒	A description of any assumptions or corrections, such as tests of normality and adjustment for multiple comparisons
☐	☒	A full description of the statistical parameters including central tendency (e.g. means) or other basic estimates (e.g. regression coefficient) AND variation (e.g. standard deviation) or associated estimates of uncertainty (e.g. confidence intervals)
☐	☒	For null hypothesis testing, the test statistic (e.g. *F*, *t*, *r*) with confidence intervals, effect sizes, degrees of freedom and *P* value noted<br>*Give P values as exact values whenever suitable.*
☒	☐	For Bayesian analysis, information on the choice of priors and Markov chain Monte Carlo settings
☐	☒	For hierarchical and complex designs, identification of the appropriate level for tests and full reporting of outcomes
☒	☐	Estimates of effect sizes (e.g. Cohen's *d*, Pearson's *r*), indicating how they were calculated

Our web collection on statistics for biologists contains articles on many of the points above.

Software and code

Policy information about availability of computer code

Data collection	Datasets were manually downloaded from public repositories such as NCBI GEO and ProteomeXChange.
Data analysis	A Python pipeline was developed and used to analyze data in this study. This pipeline, named RP3, was included in a .zip file during manuscript submission and will be made available as an open-source tool on GitHub if the manuscript is accepted for publication. Custom Python scripts used in the study are also included.<br>Additionally, we used many open-source tools to analyze the datasets:<br>- GTFtoFasta (Martinez et al., 2020)<br>- MSFragger (v3.5)<br>- Percolator (v3.06.1)<br>- FastX Toolkit (v0.0.13)<br>- Blastp (v2.12.0+)<br>- tBlastn (v2.12.0+)<br>- featureCounts (v1.6.3)<br>- STAR (v2.3.5a)<br>- samtools (v1.13)<br>- bedtools (v2.30.0)<br>- MAFFT (v7.490)<br>- RapidPeptideGenerator (rpg) (v2.0.1)<br>- MSBooster (v1.2.1)<br><br>As well as the following Python packages. These were either used in the pipeline or during custom analyses.

- Bio (v1.5.9)
- biopython (v1.81)
- matplotlib (v3.7.1)
- nheatmap (v0.1.4)
- numpy (v1.24.3)
- pandas (v2.0.3)
- pyteomics (v4.6)
- scikit_learn (v1.2.2)
- scikit_posthocs (v0.7.0)
- scipy (v1.11.2)
- seaborn (v0.12.2)
- spectrum_utils (v0.4.2)
- statannotations (v0.5.0)
- statsmodels (v0.14.0)
- venn (v0.1.3)
- pycircos (v0.3.0)

For manuscripts utilizing custom algorithms or software that are central to the research but not yet described in published literature, software must be made available to editors and reviewers. We strongly encourage code deposition in a community repository (e.g. GitHub). See the Nature Portfolio guidelines for submitting code & software for further information.

Data

Policy information about availability of data

All manuscripts must include a data availability statement. This statement should provide the following information, where applicable:

- Accession codes, unique identifiers, or web links for publicly available datasets
- A description of any restrictions on data availability
- For clinical datasets or third party data, please ensure that the statement adheres to our policy

Raw data from mouse Ribo-Seq and mass spectrometry experiments used in this study were previously generated by Martinez et al. (2022) 25 and downloaded from Gene Expression Omnibus (GEO)under accession code GSE198109 [https://0-www-ncbi-nlm-nih-gov.brum.beds.ac.uk/geo/query/acc.cgi?acc=GSE198107] and MassIVE under accession code MSV000089022 [https://massive.ucsd.edu/ProteoSAFe/dataset.jsp?task=220e5ba9df934a34a5eff5dc93081a7c], respectively. smORF sequences previously identified by Ribo-Seq and raw Ribo-Seq data were obtained from Martinez et al (2020)2 under GEO accession GSE125218 [https://www.ncbi.nlm.nih.gov/geo/query/acc.cgi?acc=GSE125218]. HLA peptidomics datasets were previously generated by Bassani-Sternberg (2015)(Ref. 38) and are available at proteomeXchange under accession PXD000394 [https://proteomecentral.proteomexchange.org/cgi/GetDataset?ID=PXD000394]. Information regarding novel microproteins identified in this study is available in Supplementary Data 1 and 2. Source data are provided with this paper.

Research involving human participants, their data, or biological material

Policy information about studies with human participants or human data. See also policy information about sex, gender (identity/presentation), and sexual orientation and race, ethnicity and racism.

Reporting on sex and gender	No human participants were involved in this study. All datasets were collected from previous studies that focused either on cell lines or mice.
Reporting on race, ethnicity, or other socially relevant groupings	No human participants were involved in this study. All datasets were collected from previous studies that focused either on cell lines or mice.
Population characteristics	No human participants were involved in this study. All datasets were collected from previous studies that focused either on cell lines or mice.
Recruitment	No human participants were involved in this study. All datasets were collected from previous studies that focused either on cell lines or mice.
Ethics oversight	*Identify the organization(s) that approved the study protocol.*

Note that full information on the approval of the study protocol must also be provided in the manuscript.

Field-specific reporting

Please select the one below that is the best fit for your research. If you are not sure, read the appropriate sections before making your selection.

☒ Life sciences ☐ Behavioural & social sciences ☐ Ecological, evolutionary & environmental sciences

For a reference copy of the document with all sections, see nature.com/documents/nr-reporting-summary-flat.pdf

Life sciences study design

All studies must disclose on these points even when the disclosure is negative.

Sample size	Sample sizes are specified in the text based on how many microproteins were identified using each dataset. All comparisons included all microproteins coming from Ribo-Seq or Proteogenomics pipelines.
Data exclusions	Quality control was performed prior to analyzing sequencing data. No other data exclusions were made.
Replication	Datasets were run through our pipeline more than once to ensure reproducibility.
Randomization	Not applicable. Datasets were previously generated and reanalyzed in this study using the same group convention to ensure consistency in data handling.
Blinding	Not applicable. Datasets were previously generated and reanalyzed in this study using the same group convention to ensure consistency in data handling.

Reporting for specific materials, systems and methods

We require information from authors about some types of materials, experimental systems and methods used in many studies. Here, indicate whether each material, system or method listed is relevant to your study. If you are not sure if a list item applies to your research, read the appropriate section before selecting a response.

Materials & experimental systems

n/a	Involved in the study
☒	☐ Antibodies
☒	☐ Eukaryotic cell lines
☒	☐ Palaeontology and archaeology
☒	☐ Animals and other organisms
☒	☐ Clinical data
☒	☐ Dual use research of concern
☒	☐ Plants

Methods

n/a	Involved in the study
☒	☐ ChIP-seq
☒	☐ Flow cytometry
☒	☐ MRI-based neuroimaging

Plants

Seed stocks	*Report on the source of all seed stocks or other plant material used. If applicable, state the seed stock centre and catalogue number. If plant specimens were collected from the field, describe the collection location, date and sampling procedures.*
Novel plant genotypes	*Describe the methods by which all novel plant genotypes were produced. This includes those generated by transgenic approaches, gene editing, chemical/radiation-based mutagenesis and hybridization. For transgenic lines, describe the transformation method, the number of independent lines analyzed and the generation upon which experiments were performed. For gene-edited lines, describe the editor used, the endogenous sequence targeted for editing, the targeting guide RNA sequence (if applicable) and how the editor was applied.*
Authentication	*Describe any authentication procedures for each seed stock used or novel genotype generated. Describe any experiments used to assess the effect of a mutation and, where applicable, how potential secondary effects (e.g. second site T-DNA insertions, mosiacism, off-target gene editing) were examined.*
