# Supplementary material for: Rp3: Ribosome profiling-assisted proteogenomics improves coverage and confidence during microprotein discovery
Source: Nat Commun. 2024 Aug 9;15:6839. doi: 10.1038/s41467-024-50301-4 (PMC11316118; doi:10.1038/s41467-024-50301-4)
Supplement: Supplementary file 3 — Description of Additional Supplementary Files [file 41467_2024_50301_MOESM3_ESM.pdf]

File name: Supplementary Data 1.xlsx
Description: Sheet 1: Microproteins identified by Rp3 from mouse adipose tissues. Columns: **microprotein**: microprotein identifier; **sequence**: microprotein amino acid sequence; **conservation**: organisms genomes where the microprotein is conserved; **num_species_conserved:** number of species with sequence conservation for that microprotein; **Ribo-seq mapping group**: mapping group for that microprotein based on our classification (MM, MM_Amb, Amb, No coverage); **MS Spectral counts**: total number of spectral counts that do not match canonical proteins. They might still match predicted microproteins; **true UTPs**: sequence of peptides that match only a single predicted microprotein**; MS peptides**: sequence of mass spectrometry peptides; **num true_UTPs**: number of peptides that match only a single predicted microprotein; **smorf_type**: smORF type based on the regions they overlap in the Ensembl annotation; **overlapped_gene**: gene overlapped by the smORF in the Ensembl annotation
Sheet 2: Microproteins identified by PRICE for the mouse adipose tissue datasets in fasta format.
Sheet 3: Microproteins identified by Ribocode for the mouse adipose tissue datasets in fasta format.

File name: Supplementary Data 2.xlsx
Description: Sheet 1: Microproteins identified by Rp3 from HLA peptidomics datasets. Columns: **microprotein**: microprotein identifier; **sequence**: microprotein amino acid sequence; **conservation**: organisms genomes where the microprotein is conserved; **num_species_conserved:** number of species with sequence conservation for that microprotein; **Ribo-seq mapping group**: mapping group for that microprotein based on our classification (MM, MM_Amb, Amb, No coverage); **MS Spectral counts**: total number of spectral counts that do not match canonical proteins. They might still match predicted microproteins; **true UTPs**: sequence of peptides that match only a single predicted microprotein**; MS peptides**: sequence of mass spectrometry peptides; **num true_UTPs**: number of peptides that match only a single predicted microprotein; **smorf_type**: smORF type based on the regions they overlap in the Ensembl annotation; **overlapped_gene**: gene overlapped by the smORF in the Ensembl annotation
Sheet 2: Microproteins identified by PRICE for the HLA peptidomics datasets in fasta format.
Sheet 3: Microproteins identified by Ribocode for the HLA peptidomics datasets in fasta format.

File name: Supplementary Data 3.xlsx
Description: Sheet 1 contains the content of a GTF file for smORFs identified with Rp3 for the mouse datasets. Sheet 2 contains the content of a GTF file for smORFs identified with Rp3 for the human datasets.
